# A customizable wireless potentiostat for assessing Ni(OH)_2_ decorated vertically aligned MoS_2_ thin films for electrochemical sensing of dopamine†

**DOI:** 10.1039/d4na00914b

**Published:** 2025-01-08

**Authors:** Topias Järvinen, Olli Pitkänen, Tomi Laurila, Minna Mannerkorpi, Simo Saarakkala, Krisztian Kordas

**Affiliations:** a Microelectronics Research Unit, Faculty of Information Technology and Electrical Engineering, University of Oulu PO Box 4500 90014 Finland topias.jarvinen@oulu.fi; b Department of Electrical Engineering and Automation, School of Electrical Engineering, Aalto University PO Box 13500, 00076 Aalto Finland; c Research Unit of Health Sciences and Technology, Faculty of Medicine, University of Oulu PO Box 5000 90014 Finland

## Abstract

In this study, we show that on-chip grown, vertically aligned MoS_2_ films that are decorated with Ni(OH)_2_ catalyst are suitable materials to be applied as working electrodes in electrochemical sensing. The constructed sensors display a highly repeatable response to dopamine, used as a model analyte, in a large dynamic range from 1 μM to 1 mM with a theoretical detection limit of 0.1 μM. In addition, to facilitate practical implementation of the sensor chips, we also demonstrate a low power wireless cyber-physical system that we designed and accommodated for cyclic voltammetry measurements. The developed cost-effective and portable instrument enables straightforward data acquisition, transfer and visualization through an Android mobile interface, and has an accuracy comparable to reference analysis of our sensors using a commercial table-top laboratory potentiostat.

## Abstract 2

Electrochemical detection of dopamine with MoS_2_–Ni(OH)_2_ thin films facilitated by a wireless, customizable and portable potentiostat.

## Graphical abstract

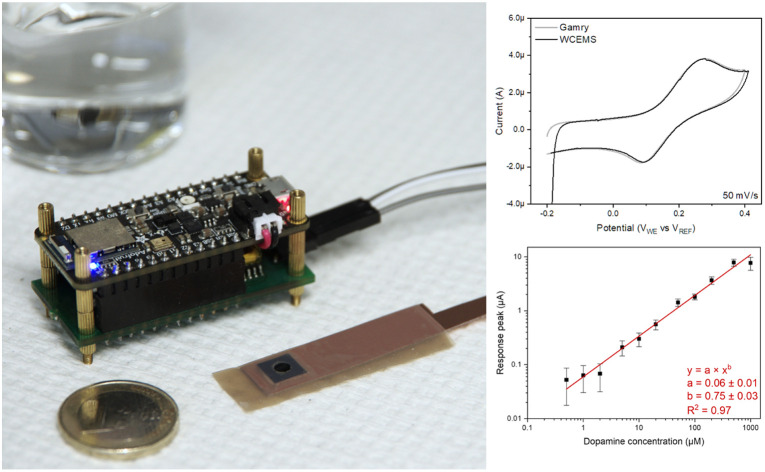

## Introduction

Transition metal dichalcogenides (TMDs) are a class of 2D materials featuring a thin, layered structure, in which an atomic metal sheet is sandwiched between two sheets of chalcogenide atoms. The strong covalent bonds between these sheets give the layer great mechanical strength, whereas the weak van der Waals interactions between the layers allow for easy exfoliation. One of the most prominent TMDs, MoS_2_, is known for its exceptional structural, electronic, optical and physico-chemical properties, which have been exploited in multitudes of applications such as field effect transistors,^1^ memristors,^2^ photodetectors,^3,4^ catalytic and photocatalytic converters,^5,6^ energy storage^7,8^ and environmental sensing using both resistive and electrochemical devices.^9–13^ The relatively easy reversible oxidation and subsequent reduction of the Mo^4+^ cations along with the presence of vacancies in the edge positions of the MoS_2_ lattice provide excellent centers for reactions between the surface and adsorbed analytes,^13–15^ thus in catalytic and electrochemical applications it appears to be a reasonable approach to orient the basal planes perpendicular to the surface, thus exposing the edge sites for any surrounding medium.^16–19^

Several different methods have been explored to synthesize various forms of MoS_2_. Powders and their suspensions in solvents may be achieved by exfoliation techniques.^20,21^ Nanoflowers, and other self-organized 3D nanomaterials are routinely produced by solvothermal methods.^22–24^ Physical^25^ and chemical vapor deposition routes^26,27^ are preferred when thin films are the subject of interest, but also sulfurization of Mo thin films supported on substrates has gained attention during the past decade.^10,28,29^ Interestingly, despite the availability of these latter methods for facile and straightforward immobilization of MoS_2_ on surfaces, electrochemical sensors are typically prepared by drop-casting or screen-printing of the exfoliated and dispersed nanomaterials onto *e.g.* glassy carbon to produce working electrodes. Although MoS_2_ itself shows electrocatalytic activity for *e.g.*, uric acid and glucose detection,^22,30,31^ to enhance the sensitivity and selectivity of the sensors, catalytic metal nanoparticles such as Ni, Cu, Pt or Au,^32–35^ reduced graphene oxide^36,37^ as well as linked functional groups^38^ and enzymes^39^ are often applied. Table 1 lists a compilation of state-of-art electrochemical sensors based on metal decorated MoS_2_.

**Table 1:** Compilation of recent electrochemical sensors based on MoS_2_ with metallic catalysts

Material	Analyte	Dynamic range (μM)	Limit of detection (μM)	References
On-chip vertically oriented MoS_2_ thin film decorated with Ni(OH)_2_	Dopamine	1–1000	0.1	This work
Ni(OH)_2_–MoS_2_ nanocomposite drop-casted onto GCE	Dopamine/α-lipoic acid	0.75–95/1–75	0.056/0.051	40
Ni single-atom decorated MoS_2_ nanosheets drop-casted onto GCE	Dopamine	1 × 10^−6^ to 1 × 10^3^	1 × 10^−6^	41
PtNi bimetallic nanoparticles loaded MoS_2_ nanosheets drop-casted onto GCE	Dopamine/uric acid	0.5–150/0.5–600	0.1/0.1	42
MoS_2_ electrodeposited on PGS doped with single Mn atoms	Dopamine	5 × 10^−5^ to 50	5 × 10^−5^	43
Au nanoparticle-decorated MoS_2_ nanosheets drop-casted onto GCE	Dopamine	0.1–200	0.08	44
NiO/MoS_2_ nanocomposite drop-casted onto GCE	Glucose	10–1 × 10^4^	1.62	35
Ni nanoparticle-functionalized MoS_2_ nanosheet drop-casted onto GCE	Glucose	0–4 × 10^3^	0.31	45
Ni-doped MoS_2_ nanoparticles on reduced graphene oxide drop-casted onto GCE	Glucose	5–8.2 × 10^3^	2.7	46
Ni nanosheet/MoS_2_ nanosheet composite drop-casted onto GCE	Nitrite	5–800	2.48	47
MoS_2_/Ni metal organic framework hybrid nanosheets drop-casted on SPGE	4-Aminophenol	0.1–600	0.04	48

In our approach, we use the sulfurization process of sputtered Mo metal films similar to that we reported in previous studies^10,49,50^ to produce electrochemical sensor chips. To enhance the redox reactions, the surface of MoS_2_ is decorated with a ∼15 Å layer of Ni followed by a short annealing resulting in NiOH modified MoS_2_.

While hundreds of papers are published yearly on electrochemical sensing in conjunction with cyclic voltammetry (CV), the typical laboratory instrumentation associated with the eventual measurements is limited to table-top computer controlled potentiostats. Therefore, in recent years, there have been several different approaches for creating affordable potentiostats with distinct design directions emphasizing features such as wireless operation, cost-efficiency, form factor, open-source availability and measurement functionality/specification for the particular applications, such as evaluating novel sensor technologies.^51^ Most of such devices rely on external user interfaces and data storage provided *i.e.* by a smartphone or computer.^52–62^ Some utilize a modular approach, incorporating oscilloscope for signal analysis^62^ or adding optical, spectrophotometric detection alongside the electrochemical measurements of a traditional tabletop potentiostat.^63^ In comparison with the potentiostat designs (Table 2), our present study, denominated as Wireless Customizable Electrochemical Measurement System (WCEMS), was designed with emphasis on small footprint and portability, wireless operation, and flexibility to adapt diverse types of sensors and experimental scenarios. The 14-bit nominal sampling accuracy can be extended in practice with four different current ranges, which are switched automatically according to the specified measurement parameters. The dimensions of WCEMS are among the smallest wireless potentiostat designs with comparable sampling accuracy, while retaining the configurable current range as in much larger ABE-Stat reported by Jenkins *et al.*^64^

**Table 2:** Comparison of potentiostat specifications

Device	Connectivity	User interface	Measurement techniques	Voltage range (mV)	Current range(s)	Sampling accuracy	Reported cost	Power source	Dimensions (mm)	Open source	Portable
WCEMS (this work)	BLE	Smart phone	CV	±1500	±1500/150/15/1.5 μA	14-Bit (16-bit ADC)	<100 USD	Li-ion battery	23 × 56 × 26	Yes	Yes
				±1000 calibrated							
CheapStat^52^	USB/serial	Computer/LCD	ACV, CV, LSV, SWV	±990	±100 nA, ±10 μA	12-Bit	80 USD	2 × AA battery/USB	140 × 66 × 28	Yes	No
Dstat^54^	USB/serial	Computer/LCD	CA, CV, DPV, SWV, POT	±1500	7	24-Bit	120 CAD	USB	80 × 80	Yes	No
UWED^56^	BLE	Smart phone	CA, CV, DPV, POT, SWV	±1500	±180 μA	10-Bit	60 USD	Rechargeable battery	∼34 × 51	Yes	Yes
KAUSTat^65^	BLE	Smart phone	CA, CV	Not reported	±5–500 μA	12-Bit	Not reported	Lithium cell battery	30 × 54	Yes	Yes
ABE-Stat^64^	Bluetooth, Wi-Fi	Smart phone	CV, DPV, EIS	±1650	±100 pA to 1.65 mA	24-Bit	105 USD	Lithium battery	74 × 89	Yes	Yes
Xu *et al.*^53^	Bluetooth	Smart phone	CA, CV, DPV	Not reported	Not reported	12-Bit	10 USD	Li-ion battery	70 × 40 × 20	No	Yes
PolArStat^60^	USB/serial	Computer	CA, CV	±3300	±13.75 mA	16-Bit	36.38 €	USB	∼100 × 73	Yes	No
PassStat 2.2 ^61^	Bluetooth	Smart phone	CV, SWV	±2400	Not reported	16-Bit	70 €	Li-ion battery	∼99 × 81	Yes	Yes
Gamry 600+ Reference	USB	Computer	CA, CV, DPV, EIS, POT, SWV *etc.*	±11 000 mV	11 (60 pA to 600 mA)	>20-Bit	∼13 500 USD	220 VAC	90 × 270 × 190	No	No

The device, powered by a Li-ion battery, is capable of independent operation while measurements are set up with a user interface on an Android mobile device. While only cyclic voltammetry was implemented and evaluated within this work, the electronics and software design principles allow for straightforward additions to the measurement options such as square wave voltammetry (SWV) and differential pulse voltammetry (DPV) as well as modifications to the electronics design. To demonstrate the proof-of-concept of our design, we assessed the sensing capabilities of our on-chip NiOH decorated MoS_2_ electrodes performed on a model analyte, dopamine, using CV. To validate the results obtained with WCEMS, we performed reference measurements using a high-end commercial potentiostat.

## Results

The effect of Ni decoration on the surface structure of MoS_2_ films is analyzed with atomic force microscopy (AFM). Three different maps are collected from pristine MoS_2_ as well as Ni-decorated films before and after annealing, Fig. S1.† The root mean square (RMS) roughness (Sq) values (1.78–1.86, 2.30–2.14 and 2.38–2.41 nm for MoS_2_, MoS_2_ + Ni non-annealed and MoS_2_ + Ni annealed, respectively) show slight increase, which suggests agglomeration of Ni during the annealing process.

**Fig. 1:**
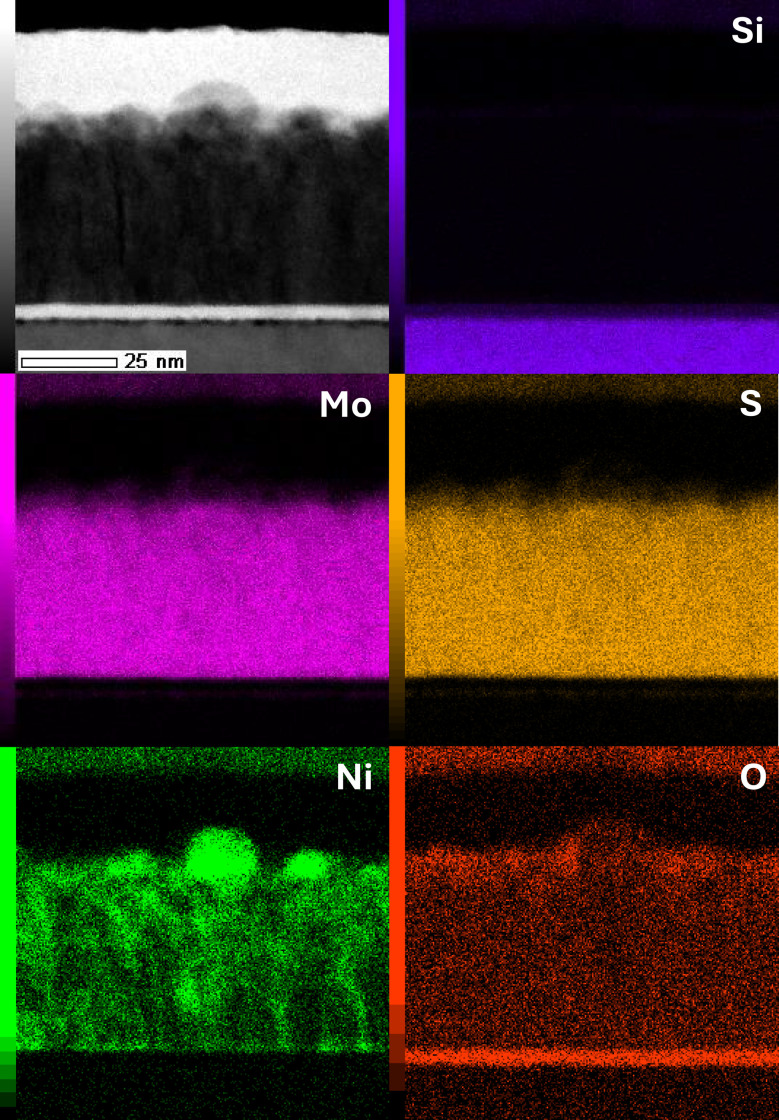
EDX maps of the MoS_2_–Ni(OH)_2_ films show the deposited nickel as particles on top of the TMD film. In addition, some nickel is diffused into the TMD film.

Energy-dispersive X-ray spectroscopy (EDX) mapping of cross section of the MoS_2_ + Ni annealed sample, Fig. 1, shows a very homogeneous MoS_2_ film with thickness of approximately 45 nm as well as several nickel particles on the top of the film. The surface of these particles shows some oxidation. It can be seen as well that some of the nickel has diffused into the TMD film. In ESI (Fig. S2†), average atomic percentages are analyzed from the MoS_2_ film and from a single particle on the surface. The film has some excess sulfur with a metal to sulfur ratio of 1 : 2.6 whereas nickel contents within the film are 6.5 at%. On the other hand, EDX of the particle shows mostly nickel and oxygen contents (48.2 and 23.2 at%, respectively). A higher resolution TEM image of the cross section depicting the Ni particles on top of the vertical layered structure is provided in the ESI (Fig. S3†).

**Fig. 2:**
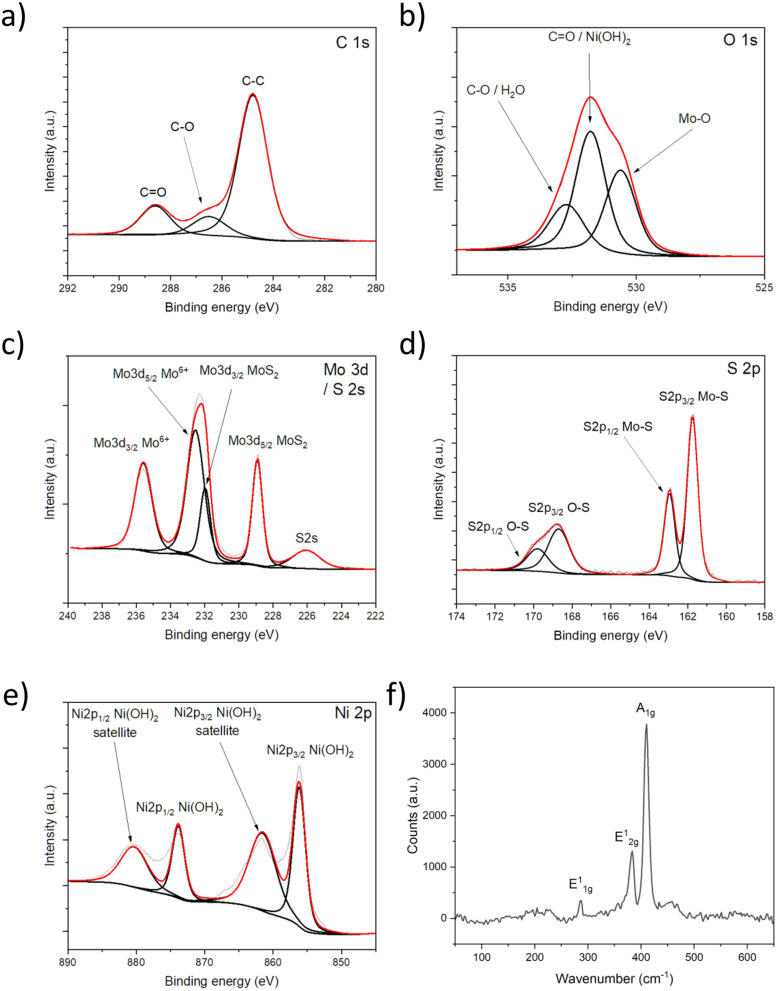
XPS (a)–(e) and Raman (f) characterization of the MoS_2_–Ni(OH)_2_ thin films.

Raman spectroscopy, carried out over six sulfurization batches, proves that the process is very repeatable and produces homogeneous films. A representative, noise-filtered spectrum is shown in Fig. 2f, while a comprehensive set of spectra is available in Fig. S4.† The main peaks, E^1^_2g_ at 383 cm^−1^ (in-plane mode) and A_1g_ at 410 cm^−1^ (out-of-plane mode) correspond to bulk MoS_2_. This is expected as the film formed by the vertically oriented planes has a thickness of ∼100 nm according to previous studies.^49,50,66^ Vertical orientation is implied by the stark difference between the peak intensities.^18,67,68^ Furthermore, most of the measured spectra show the so-called forbidden E^1^_1g_ peak at around 285 cm^−1^, which is typically invisible for laterally oriented crystals, is now handily detected from the exposed edge planes on the topmost surface of the vertically oriented MoS_2_ films.^69^

X-ray photoelectron spectroscopy (XPS) analysis is performed on a Ni decorated and annealed MoS_2_ sample (Fig. 2a–e) to explore the chemical composition of the surface. The C 1s peak components (Fig. 2a) indicate only adventitious carbon (C–C at 284.8 eV, C–O at 286.5 and C=O at 288.6 eV) on the surface. The resolved O 1s peak (Fig. 2b) shows Mo–O bond at 530.6 eV (indicating partial oxidation of the MoS_2_ lattice); C=O and/or Ni(OH)_2_ at 531.8 eV, and C–O and/or surface water at 532.7 eV.^40,70,71^Fig. 2c shows the deconvoluted Mo 3d spectrum, in which the peak at 226.1 eV is attributed to S 2s, the doublet at 228.9/232.0 eV refers to Mo^4+^*i.e.*, to MoS_2_, whereas the other doublet at 232.5/235.6 eV corresponds to Mo^6+^*i.e.*, to Mo–O bonds. In the S 2p spectrum (Fig. 2d), the Mo–S bond is indicated by the doublet at 161.7/162.9 eV,^50,72^ whereas the other two peaks at 168.7 eV and 169.8 eV are associated with oxidation products, most likely sulfate.^50,73^ The resolved Ni 2p spectrum (Fig. 2e) shows a doublet at 856.2 eV and 873.9 eV and corresponding satellites at 861.7 eV and 880.5 eV indicating that mostly Ni(OH)_2_ is present on the surface, which is reasonable considering the hydroxide peak in the O 1s spectrum at 531.8 eV.^74,75^ The XPS results agree with the EDX analysis, describing oxidized nickel contents on the film surface.

**Fig. 3:**
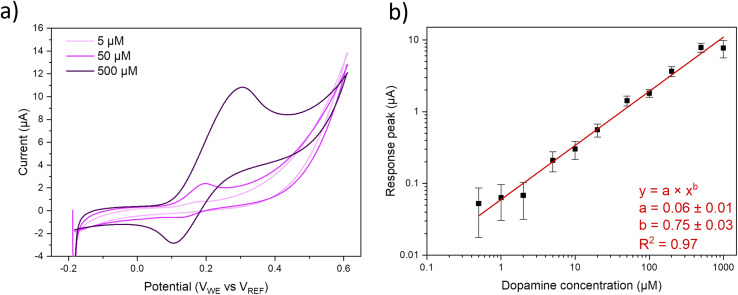
CV data acquired with WCEMS at 5, 50 and 500 μm dopamine concentrations (a). Power function fit for the response peak currents collected jointly with Gamry and WCEMS (b).

**Fig. 4:**
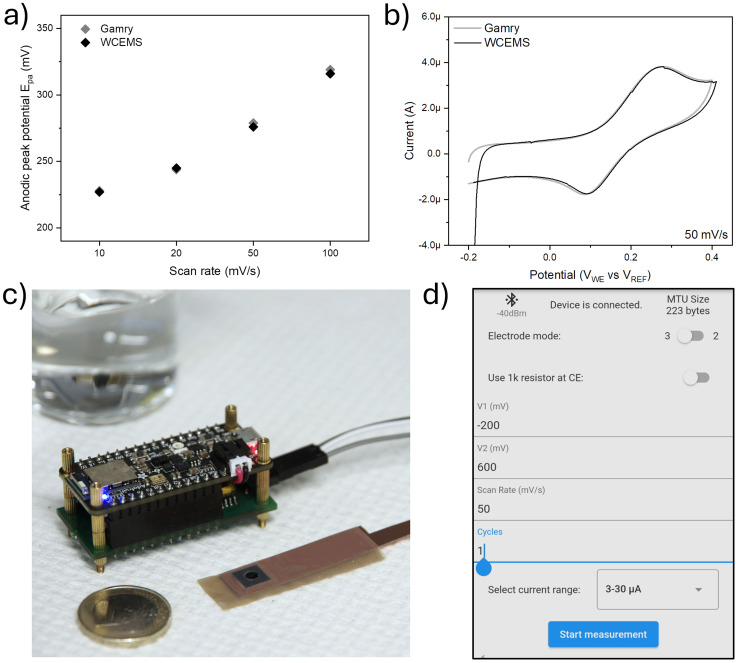
Peak potential shift with increasing scan rates at 100 μm concentration, (a) and a comparison between Gamry and WCEMS output data at the scan rate of 50 mV s^−1^ (b). WCEMS and a working electrode assembly with MoS_2_–Ni(OH)_2_ sample (c). 1 Euro coin as size reference. User interface running on android mobile device (d).

Potential windows of the pristine MoS_2_ as well as MoS_2_–Ni(OH)_2_ samples are assessed from CV scans between −2 and 2 V in phosphate buffered saline (PBS), Fig. S5a.† According to the selected threshold current of ±10 μA, the feasible potential window is approximately −1 V to 1 V *vs.* Ag/AgCl reference. Pseudocapacitances, calculated from the difference between anodic and cathodic currents^76^ measured between −0.2 and 0.8 V *vs.* Ag/AgCl in PBS are 1 and 97 μF cm^−2^ for pristine MoS_2_ and MoS_2_–Ni(OH)_2_, respectively. Fig. S5b† also shows no discernible response for dopamine in the case of pristine MoS_2_, while a clear response is observed after the introduction of Ni(OH)_2_. Measurements using the outer sphere redox probe ferrocenemethanol were conducted to investigate the electronic properties of the electrode materials, Fig. S6.† The peak separation between the anodic and cathodic peaks suggests that the electron transfer reaction on these materials is at the slow side of quasi-reversible region and thus highly dependent on the scan rate. As the scan rate increases, the peak separation expands rapidly, reaching substantial values even at 400 mV s^−1^. These observations indicate slow electron transfer kinetics for these materials. The electrochemical performance of MoS_2_–Ni(OH)_2_ is assessed with a known inner sphere redox probe, dopamine. CV was initially carried out at the range of −200 to 600 mV for concentrations from 1 up to 1000 μM including the reference measurements of pristine MoS_2_ film (Fig. S5b†) at concentrations of 10, 100 and 1000 μM which did not produce any quantifiable response. Since the background level starts to rise from 400 mV onwards, due to the oxidation of MoS_2_ and Ni(OH)_2_ deteriorating the signal-to-noise ratio (SNR), measurements at lower concentrations (100, 200 and 500 nM) were carried out at a reduced scan range of −200 to 400 mV. Typical responses at 5, 50 and 500 μM concentrations, measured with WCEMS, are shown in Fig. 3a. The calibration curve, displayed in Fig. 3b as log–log plot of response current *vs.* dopamine concentration, obeys a power fit (with exponent of 0.75 ± 0.03). The statistical analysis of the measurement data is presented in Table S1.† As indicated by the error margins of statistical analysis, the reproducibility of the measurements across different samples and fabrication batches is reasonably good, especially at higher concentrations than 2 μM. The sensitivity calculated from the linear fit of sensor data in the concentration range of 0.5 to 50 μM is 0.03 μA μM^−1^, or 0.43 μA μM^−1^ cm^−2^ considering the area of the electrode, *i.e.* 0.07 cm^2^. The theoretical limit of detection (LOD) is estimated to be 0.1 μM or better, as determined from the standard deviation of the signal (*σ* ∼ 6 × 10^−10^ A) and the sensitivity value (*S* = 0.03 μA μM^−1^) according to the definition LOD = 3.3 × *σ*/*S*. The respective limit of quantification (LOQ) is one order of magnitude higher at ∼1 μM. The kinetics of the dopamine reaction exhibit characteristics of quasi-reversibility, as shown by the shift of the anodic peak potential *E*_pa_ as a function of scan rate, Fig. 4a. The logarithm of the peak currents can be fitted linearly with logarithmic scan rates, Fig. S6,† with a slope of 0.47 which correlates well with the theoretical value of 0.5 for diffusion-controlled processes.

The results show detection of dopamine in the dynamic range of 1–1000 μM. Performance is comparable to some MoS_2_ based composite materials, such as MoS_2_/PEDOT,^77^ metal catalysts (Table 1) apart from single-atom doped MoS_2_.^41,43^ The hydroxide groups on the material surface are expected to facilitate the redox reactions of the analyte by hydrogen bonding with dopamine as it is oxidized into dopamine-*o*-quinone.^40^ The catalytic mechanism relies on the transition of proton tunneling from diabatic to adiabatic states, resulting in a subsequent reduction in the activation energy for proton transfer.^78^ While a systematic shelf-life study was not performed in our work, we would like to note that the outer-sphere redox probe ferrocenemethanol measurements (Fig. S7†) were carried out on approximately 12 months old samples indicating that rapid aging of the surface under ordinary lab conditions does not seem to be of concern. Interference measurements, Fig. S8,† performed on 1, 10 and 100 mM of glucose, 0.1, 1 and 10 μM of uric acid well as 0.1, 1 and 10 mM of ascorbic acid, show no response either uric acid or glucose. In the case of ascorbic acid, a response is observed in millimolar concentrations, overlapping with dopamine peaks. However, in case of *in vitro* measurements that the portable design of WCEMS is well suited for, the ascorbic acid decomposes relatively fast, after which the dopamine can be effectively measured.^79^ All in all, the relatively slow reaction kinetics alongside large error margins in nanomolar concentrations still call for further optimization of the on-chip MoS_2_ films *e.g.* by engineering its surface chemistry with specific ligands or with co-catalyst nanomaterials that can selectively bind to analytes and facilitate improved charge transfer.^80–83^ Furthermore, portable measurements in realistic use cases would greatly benefit from integrated counter and reference electrodes on the sensor chip as such configuration could improve not only the signal-to-noise ratio (hence resolution and limit of detection) but also the practical use of the setup.

A comparison of back-to-back measurements performed on the same sample at dopamine concentration of 100 μM with both reference device and WCEMS is shown in Fig. 4b. Additional comparisons at different scan rates between 10 and 100 mV s^−1^ are collected to Fig. S9.† The background signal deviation for WCEMS defined at 500 nM concentration (Fig. S10†) is approximately *σ* ∼ 5.3 × 10^−9^ A, which sets the LOD of the device to ∼500 nM (being about five times higher than that of reference potentiostat). Therefore, in the context of the presented study, the noise level and data resolution of the WCEMS are sufficient to assess the sensitivity of the MoS_2_–Ni(OH)_2_ for dopamine. It is worth noting that due to the increased background the lowest current range (*i.e.*, at the highest amplification) could not be used in these measurements. The jump in the beginning of the measurement data, as seen is Fig. 3 as well as Fig. S9,† is most probably caused by remaining bias in the electrochemical cell. This could be alleviated by adding a stabilization period in the software before running the voltage cycle or shorting the collector and working electrodes with an internal switch. Fig. 4c shows the WCEMS alongside a prepared working electrode and 4d depicts the user interface (UI) elements of the software. It is also important to note that the correction of the measured data was carried out on a computer (using OriginPro) according to the calibration curves, which may be also pre-processed on the smart phone in the future with a software upgrade. Furthermore, the design of the WCEMS allows for additional measurement functions such as differential and square wave voltammetries as well as chronoamperometry. The presented measurement scenario of MoS_2_–Ni(OH)_2_ in dopamine sensing did not utilize all the current ranges available, which could overestimate the noise performance at highest level of amplification. The rudimentary UI was designed to carry out the proof-of-concept measurement and has room for improvement, especially for continuous measurements and handling larger datasets.

## Experimental

### Materials

MoS_2_ thin films were produced by adopting the protocols reported previously.^10,49,50^ Specifically, p^++^ B-doped Si wafers with resistivity of <0.005 Ω cm were laser-cut halfway through from the backside into 7 × 7 mm^2^ square patterns with an LPKF ProtoLaser U3 (Nd:YVO_4_, *λ* = 355 nm, *P*_avg_ = 6 W, *f* = 40 kHz, *τ* = ∼20 ns, ∼20 μm focal spot diameter). The contact resistance between the substrate and TMD film was minimized by etching the native oxide layer of the Si substrate with buffered hydrofluoric acid. Immediately after etching, a thin Mo film (20 nm) was deposited by sputtering (Torr International PVD System) and the chips were subsequently sulfurized with 1 g of S powder (Sigma-Aldrich 215236, ≥95%) in a tube oven (Thermo Scientific Thermolyne with a quartz tube of 2′′ in diameter) at 800 °C under 400 sccm N_2_ flow for 1 hour. Each sulfurization process produced a batch of 12 sensor chips. After sulfurization, Ni with a thickness of ∼15 Å was deposited with the physical vapor deposition (PVD) system followed by annealing for 1 h at 400 °C under 400 sccm Ar flow.

The structure, physical and chemical properties of similar on-chip MoS_2_ films have been characterized extensively in previous works,^10,49,50^ which is now complemented with Raman analyses of multiple sample batches to assess the stability of the sulfurization process. The cross-section lamella of the MoS_2_–Ni(OH)_2_ film was prepared with focused ion beam (FIB, FEI Helios DualBeam) and analyzed by transmission electron microscope (TEM, JEOL JEM-2200FS EFTEM/STEM) including EDX mapping. In addition, AFM (MultiMode 8, Nanoscope V, Bruker) is used to compare the surface morphology and roughness before and after the deposition of Ni and subsequent annealing. Also, XPS analysis (Thermo Fischer Scientific ESCALAB 250Xi) is carried out to understand the chemical composition of the surface after Ni decoration and annealing.

### Sensor preparation and electrochemical measurements

The electrochemical sensors were prepared as follows. First, the native oxide from the backside of the chips was mechanically removed. Then, the chips were mounted on strips of copper-plated printed circuit board (PCB) using silver paste. Finally, the as-obtained structures were insulated with Teflon tape, sans a punched 3 mm diameter hole on the tape, confining the active sensor area to approximately 7 mm^2^. On-chip MoS_2_ films without deposited Ni were also prepared and measured as reference samples. These assemblies were then used as working electrodes in three-electrode configuration measurements in conjunction with a Pt wire (Sigma-Aldrich 267228, 99.9%) as counter and Ag/AgCl (in saturated KCl) as the reference electrodes (Radiometer Analytical XR300).

With the exception of the 1 mM ferrocenemethanol (Thermo Scientific Chemicals, 1273-86-5, 97%) outer-sphere redox probe measurements done in 1 M KCl (Sigma-Aldrich, P3911, ≥95%), 0.01 M PBS (Sigma-Aldrich SIALP3813) was used as electrolyte in all experiments including the preparation of dopamine solutions (0.1–1000 μM, dopamine hydrochloride, Sigma-Aldrich H8502, ≥95%). CV was performed with voltage scans from −200 mV up to 400–800 mV depending on the concentration range with 3 subsequent cycles performed at a scan rate of 50 mV s^−1^. N_2_ was bubbled through the electrolyte to remove oxygen as well as to provide mixing during the measurements. A minimum of three samples were measured at each concentration for assessing statistical variation. The electrochemical results were collected using either or both Gamry Reference 600+ (Gamry Instruments, Inc.) and WCEMS devices as stated in the figure captions.

### Electronics and software design of WCEMS

Adafruit Feather nRF52840 Sense (Adafruit Industries LLC) microcontroller main board was selected as the center point of the device design since it incorporates Bluetooth Low Energy (BLE) communication capabilities, reasonably small footprint and integrated Li-ion battery charger circuit in a single system. The potentiostat PCB was designed to conform with Adafruit's FeatherWing expansion card form factor and could thus be stacked with the microcontroller PCB and a Li-ion battery (400 mA h/3.7 V, Shenzhen PKCELL Battery Co., Ltd). The device dimensions are approximately 23 × 56 × 26 mm^3^ and weight of 28.3 g including the battery. The modular approach of the PCBs allows a clear separation between the microcontroller and potentiostat circuits, with only I/O pins, operating voltage and ground transferred between the boards. This makes it straightforward to upgrade either of the boards, as long as the form factor and pinouts are kept same. In addition, separating the radio frequency (RF) and battery charging components from the measurement circuit board can improve noise performance.

The electronics design for the potentiostat circuit board (Fig. S11†) can be broken down into six sections: (1) headers for connections and power delivery between potentiostat and microcontroller PCBs. (2) 16-Bit digital-to-analog (DAC) converter with 3.0 V voltage reference (AD5663 & REF193, Analog Devices Inc.) to carry out the voltage ramps. (3) An 8-channel single-pole single-throw (SPST) switch (MAX395, Analog Devices, Inc.) controls the operation of the potentiostat, such as connecting the cell electrodes, optional 1 kΩ resistor at counter electrode to dampen oscillation and the different feedback resistors of the transimpedance amplifier. The amplifier (4) has 4 gain settings between 10^3^ and 10^6^ determined with precision resistors, followed by a 4th order Sallen-Key low-pass filter stage (5) with a cutoff frequency of 10 Hz. An LTspice simulation of analog signal processing stage is presented in Fig. S12.† In the end of the signal path, a 16-bit analog-to-digital (ADC) converter (6) (ADS1118, Texas Instruments Inc.) is used to quantize the signal, with optional inputs for directly sampling the digital-to-analog converter and raw transimpedance amplifier outputs as well as the virtual ground level. All operational amplifiers (*i.e.* used in the transimpedance amplifier, low-pass filter as well as in the feedback circuit of the electrochemical cell) are MCP6022 type (Microchip Technology Inc.). The four-layer PCB incorporates two dedicated copper layers for operating voltage and ground plane. Electronics schematic and layout are implemented with KiCad 6.0.1 EDA and made available in open data repository (Zenodo).

The microcontroller is programmed with C++ using Arduino 2.2.1 IDE (Arduino S.R.L.). The program receives initial parameters over Bluetooth low energy (BLE), executes CV measurements accordingly and sends the result data back to the control device to be displayed in the user interface. The DAC output refresh frequency is set to 200 Hz while sampling frequency of ADC is determined to be twice the cutoff frequency of the low-pass filter, *i.e.* 20 Hz. The implementation of CV loop is presented in ESI,† while the rest of the codebase is available in the code repository. The user interface for Android mobile device was implemented with Flutter in Visual Studio 1.85.1 IDE (Microsoft Corporation). The interface (Fig. 4d) allows for the selection of CV parameters such as voltage range, scan rate, number of cycles and desired level of amplification. The ongoing measurement can be observed in real time and the result is saved in .csv format on the device. The codebase is made available in GitHub under MIT license. Demonstration of the measurement setup and data collection is provided in the ESI† video file.

The calibration of the WCEMS is carried out by measuring CVs with a scan window between −1000 and 1000 mV with precision resistors from 1 kΩ to 1 MΩ for each amplification level. The results (Fig. S13†) are fitted in OriginPro and corrections are calculated according to the linear fit to match the desired 1000 mV output at transimpedance amplifier at maximum of the voltammetry ramp. The respective correction factors were then applied for the collected measurement data.^84^

## Conclusions

In this study, vertically aligned MoS_2_ thin films modified with Ni(OH)_2_ were synthesized directly on Si chips and assessed as working electrodes for electrochemical sensors. AFM and XPS were carried out to investigate surface structure and chemical composition, while Raman spectroscopy verified the repeatability of the sulfurization process. The on-chip electrodes proved to be sensitive for dopamine with a dynamic current response at concentrations between 1 and 1000 μM with a theoretical limit of detection of 0.1 μM. The sensor measurements were accompanied by a custom proof-of-concept portable potentiostat design and demonstration, suitable for cyclic voltammetry measurements and wireless transmission of the data. The potentiostat performance was assessed to be comparable to the reference device in the measurements, while having the capability to perform experiments outside the laboratory. In addition, the open-source design is straightforward to modify, thus having the potential to address other measurement parameters and functions of interest. The results of this research work establish a foundation for the future development of on-chip TMD materials based electrochemical sensing and highlight the importance of incorporating co-catalyst nanomaterials to enhance electrocatalytic activity as well as to engineer chemical selectivity.

## Author contributions

TJ: conceptualization, software, data curation, formal analysis, validation, investigation, visualization, methodology, writing – original draft, writing – review & editing. OP: conceptualization, formal analysis, investigation, methodology, writing – review & editing. TL: conceptualization, formal analysis, validation, investigation, writing – review & editing. MM: investigation. SS: resources, supervision. KK: conceptualization, resources, formal analysis, supervision, funding acquisition, project administration, writing – review & editing.

## Data availability

Characterization and measurement data are available in Zenodo repository (https://doi.org/10.5281/zenodo.10808987) interlinked to a Github repository containing the WCEMS software.

## Conflicts of interest

There are no conflicts to declare.

## Supplementary Material

NA-007-D4NA00914B-s001

NA-007-D4NA00914B-s002
