# Supplementary material for: A customizable wireless potentiostat for assessing Ni(OH)2 decorated vertically aligned MoS2 thin films for electrochemical sensing of dopamine
Source: Nanoscale Adv. 2025 Jan 8;7(5):1374–83. doi: 10.1039/d4na00914b (PMC11747886; doi:10.1039/d4na00914b)
Supplement: NA-007-D4NA00914B-s001 [file NA-007-D4NA00914B-s001.pdf]

Supplementary Information (SI) for Nanoscale Advances.
This journal is © The Royal Society of Chemistry 2025

A customizable wireless potentiostat for assessing $Ni(OH)_2$ decorated vertically aligned MoS_2 thin films for electrochemical sensing of dopamine

Järvinen, T.*[1], Pitkänen, O.[1], Laurila, T.[2], Mannerkorpi, M.[3], Saarakkala, S.[3], and Kordas, K.[1]

* Corresponding author. Contact e-mail: topias.jarvinen@oulu.fi

1 Microelectronics Research Unit, Faculty of Information Technology and Electrical Engineering, University of Oulu, FI-90014 Oulu, Finland.

2 Department of Electrical Engineering and Automation, School of Electrical Engineering, Aalto University, PO Box 13500, 00076 Aalto, Finland.

3 Research Unit of Health Sciences and Technology, Faculty of Medicine, University of Oulu, POB 5000, FI-90014, Oulu, Finland.

Supplementary information

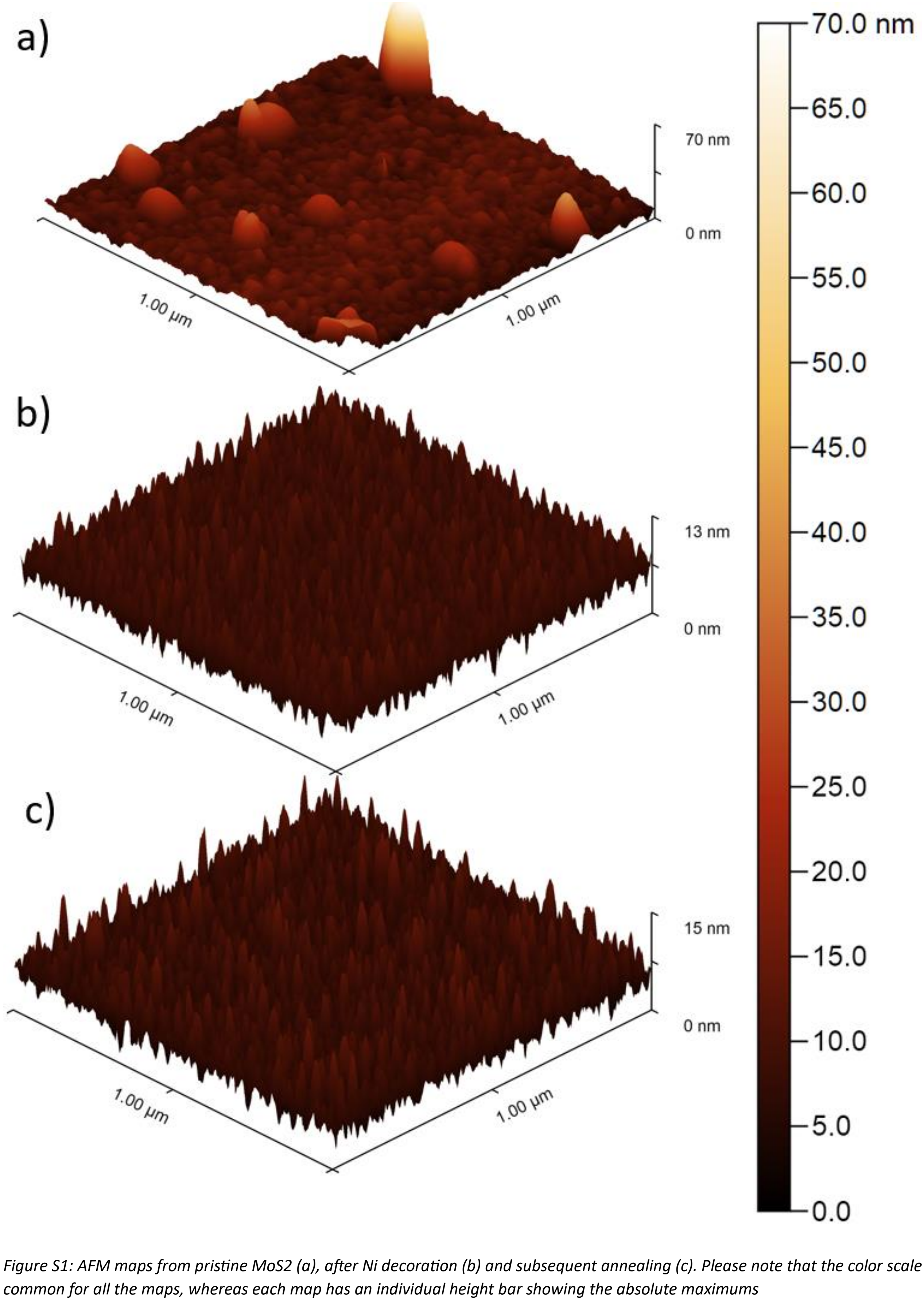

Figure S1: AFM maps from pristine MoS2 (a), after Ni decoration (b) and subsequent annealing (c). Please note that the color scale is common for all the maps, whereas each map has an individual height bar showing the absolute maximums

a)

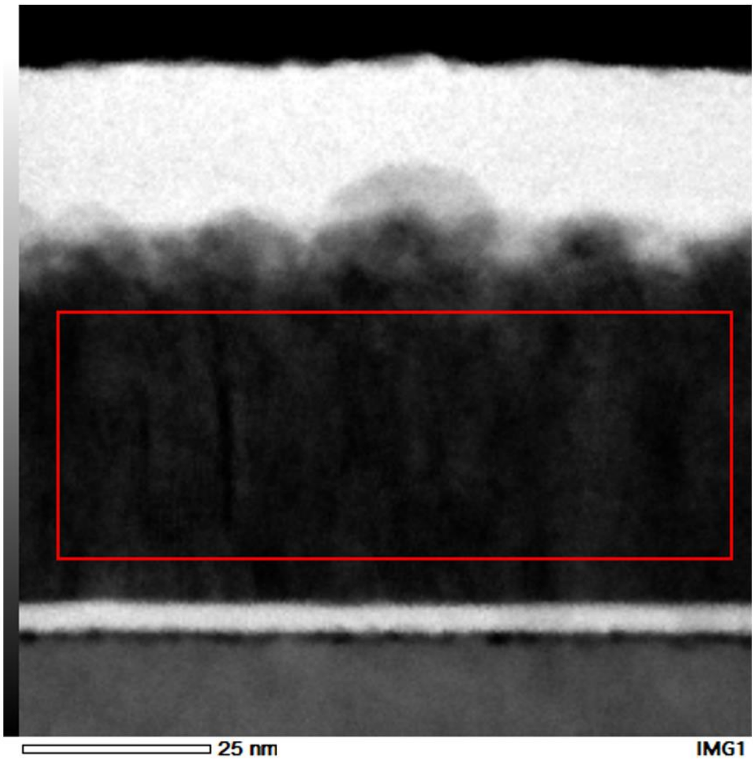

Element	Atomic percent (%)
O	4.2
Si	1.7
S	63.3
Ni	6.5
Mo	24.3
Total	100.0

b)

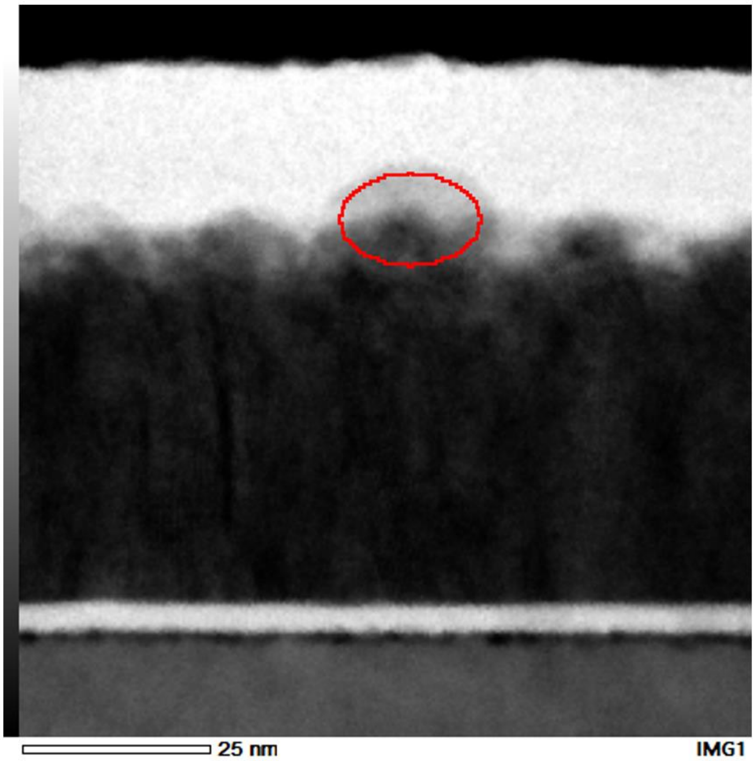

Element	Atomic percent (%)
O	23.2
Si	5.4
S	16.0
Ni	48.2
Mo	7.1
Total	100.0

Figure S2: EDX elemental quantization from the a) bulk TMD film and b) Nickel particle on top of the film

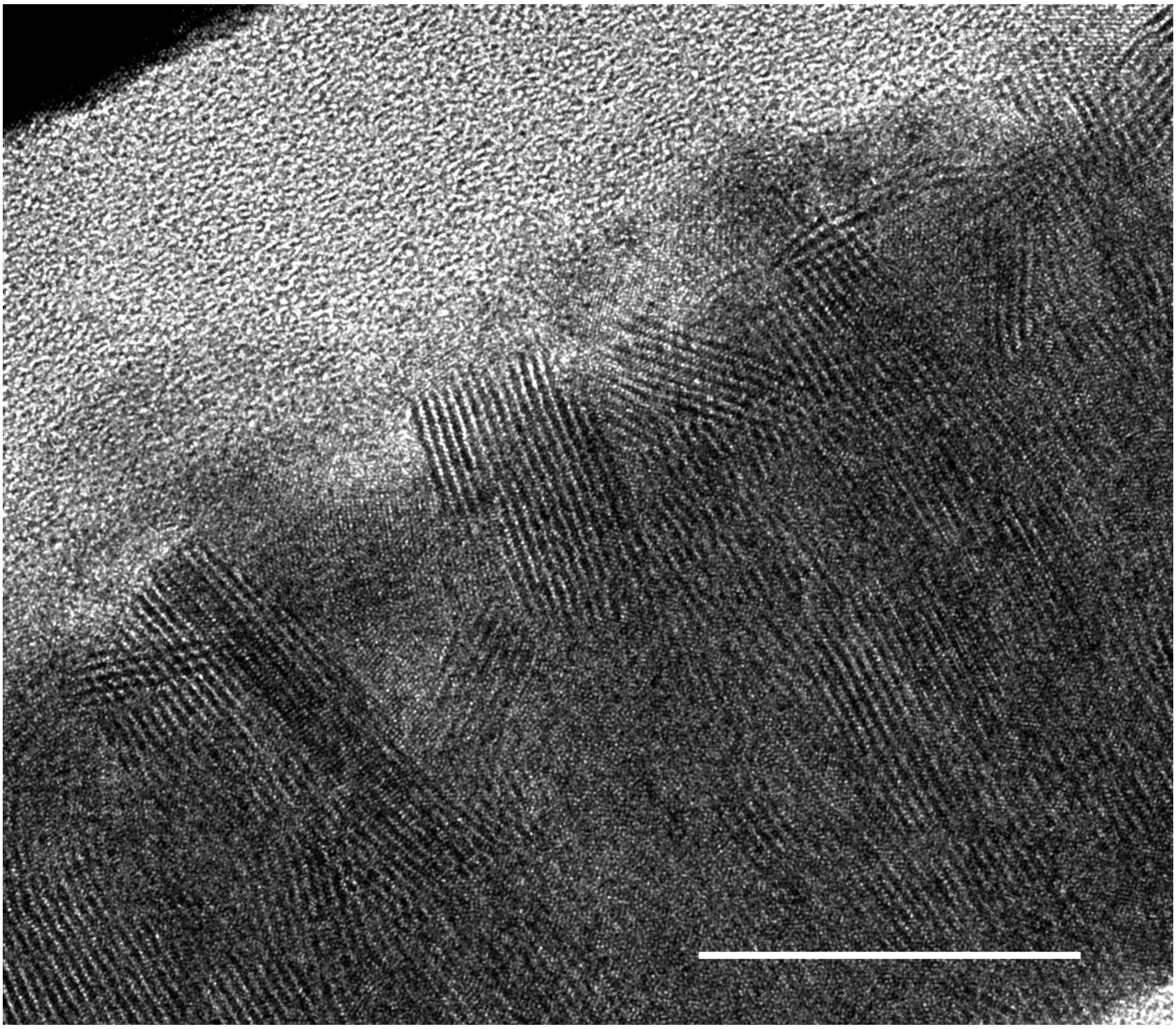

Figure S3: TEM image of the cross-section of the MoS_2-$Ni(OH)_2$ film. The nickel particles are seen on top of the vertical layered structure of the MoS_2. The scalebar is 20 nm.

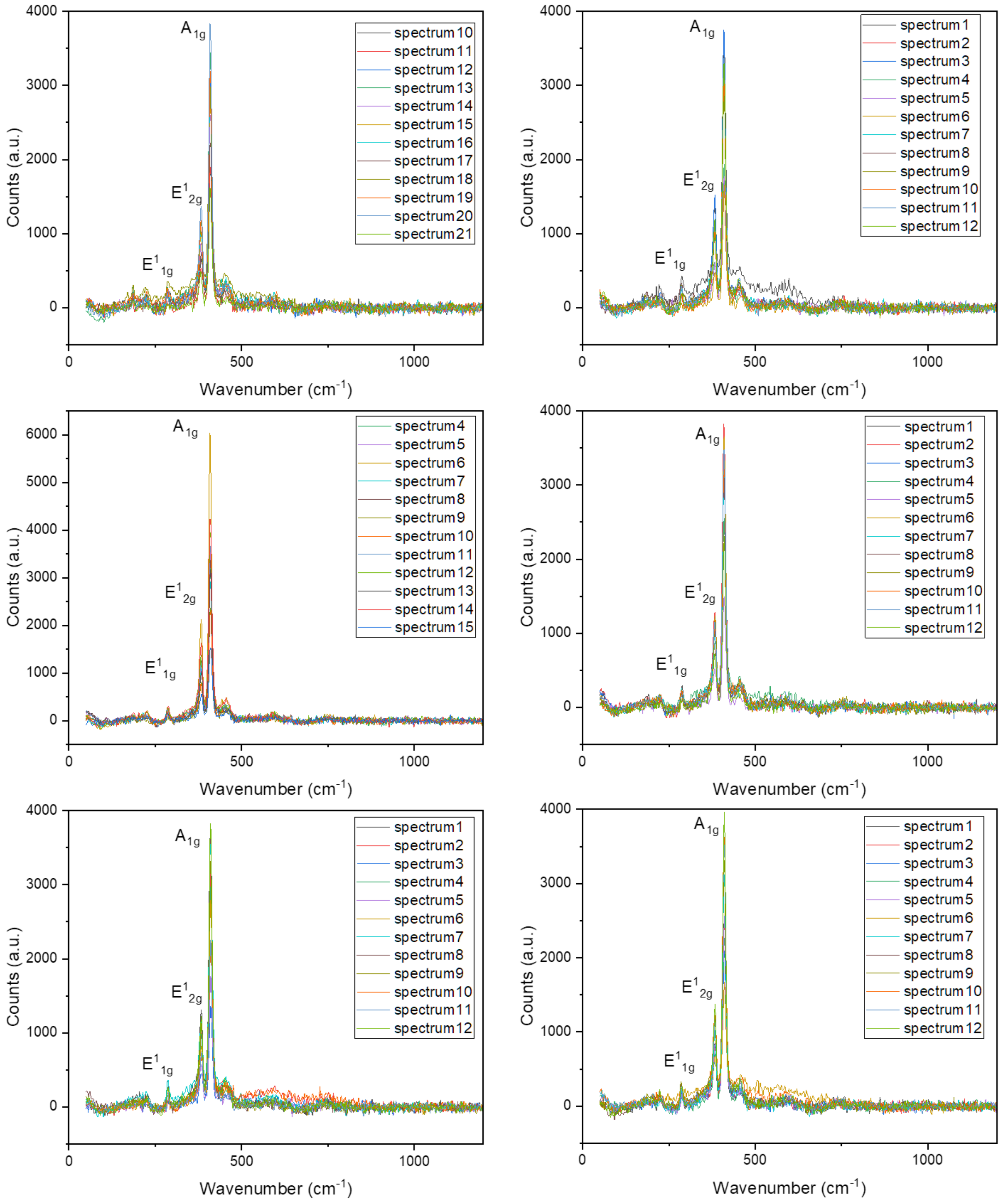

Figure S4: Raman spectra from six different sulfurization processes

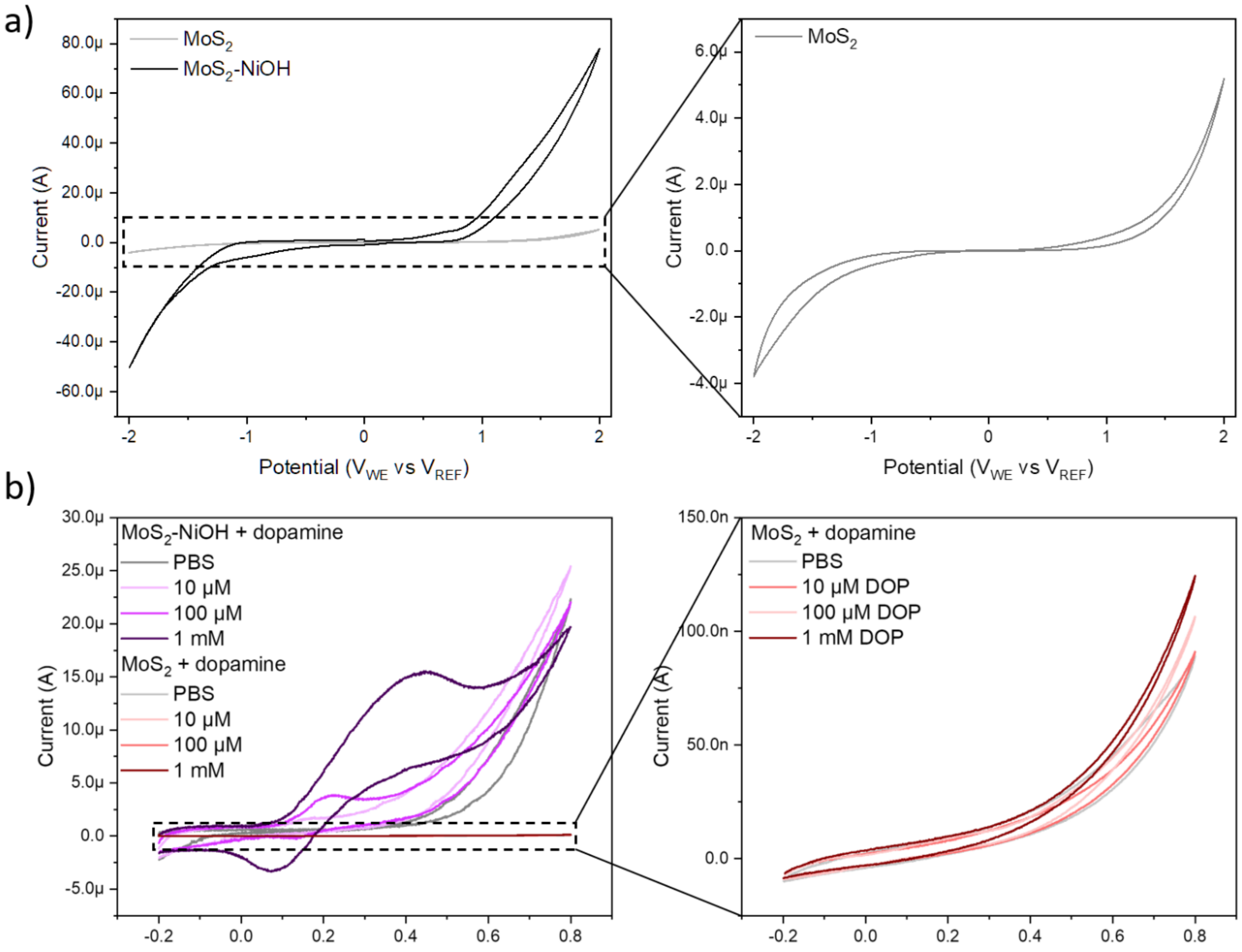

Figure S5: Comparison of potential windows between pristine MoS_2 and MoS_2-NiOH films measured from -2 to 2 V (a) and responses for 10, 100 and 1000 µM of dopamine, measured with Gamry

Table S1: Dopamine CV measurement statistics

Dopamine concentration (μM)	Samples (N)	Mean (μA)	Standard Deviation (μA)
0.5	4	0.052	0.035
1	4	0.063	0.033
2	3	0.068	0.036
5	5	0.210	0.066
10	3	0.301	0.084
20	4	0.558	0.117
50	5	1.426	0.231
100	4	1.801	0.221
200	5	3.665	0.580
500	5	7.842	1.181
1000	5	7.728	2.079

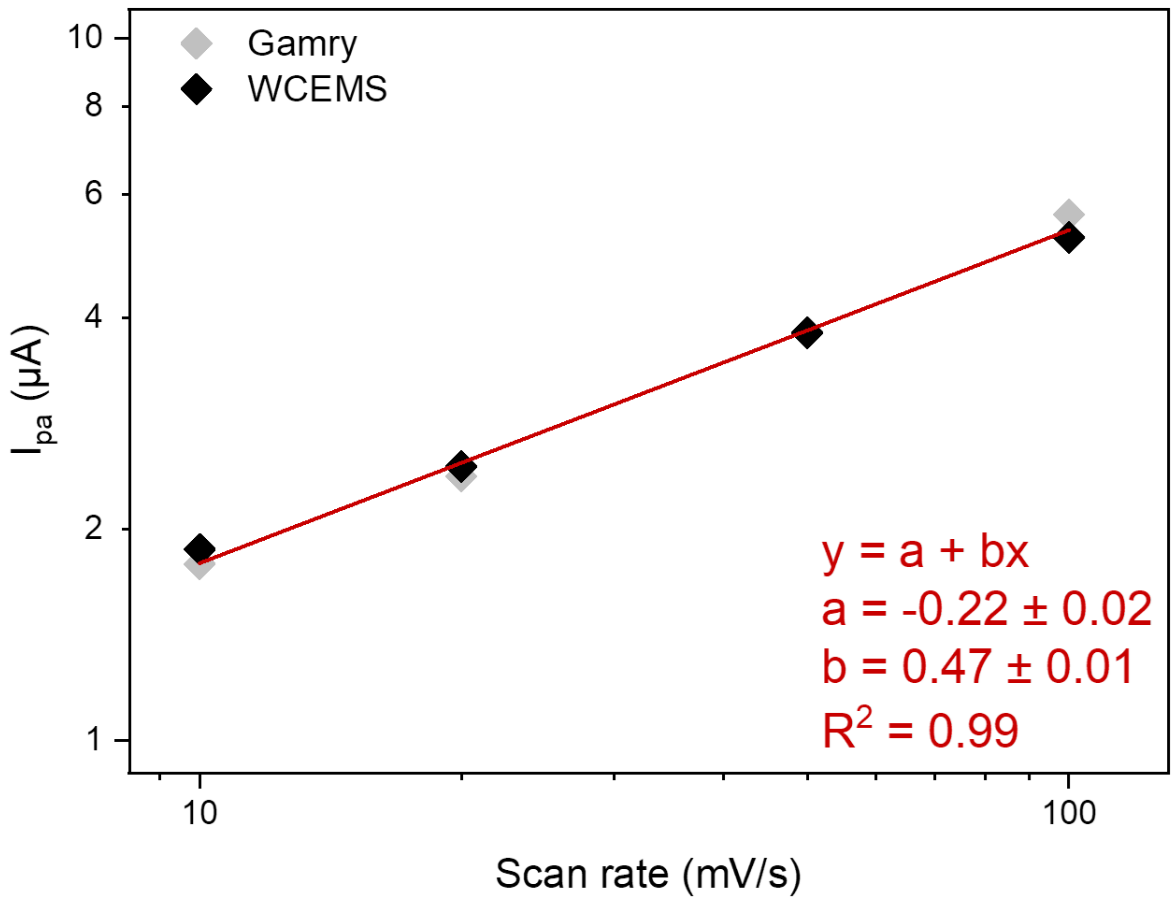

Figure S6: Anodic peak current plotted against logarithmic scan rates

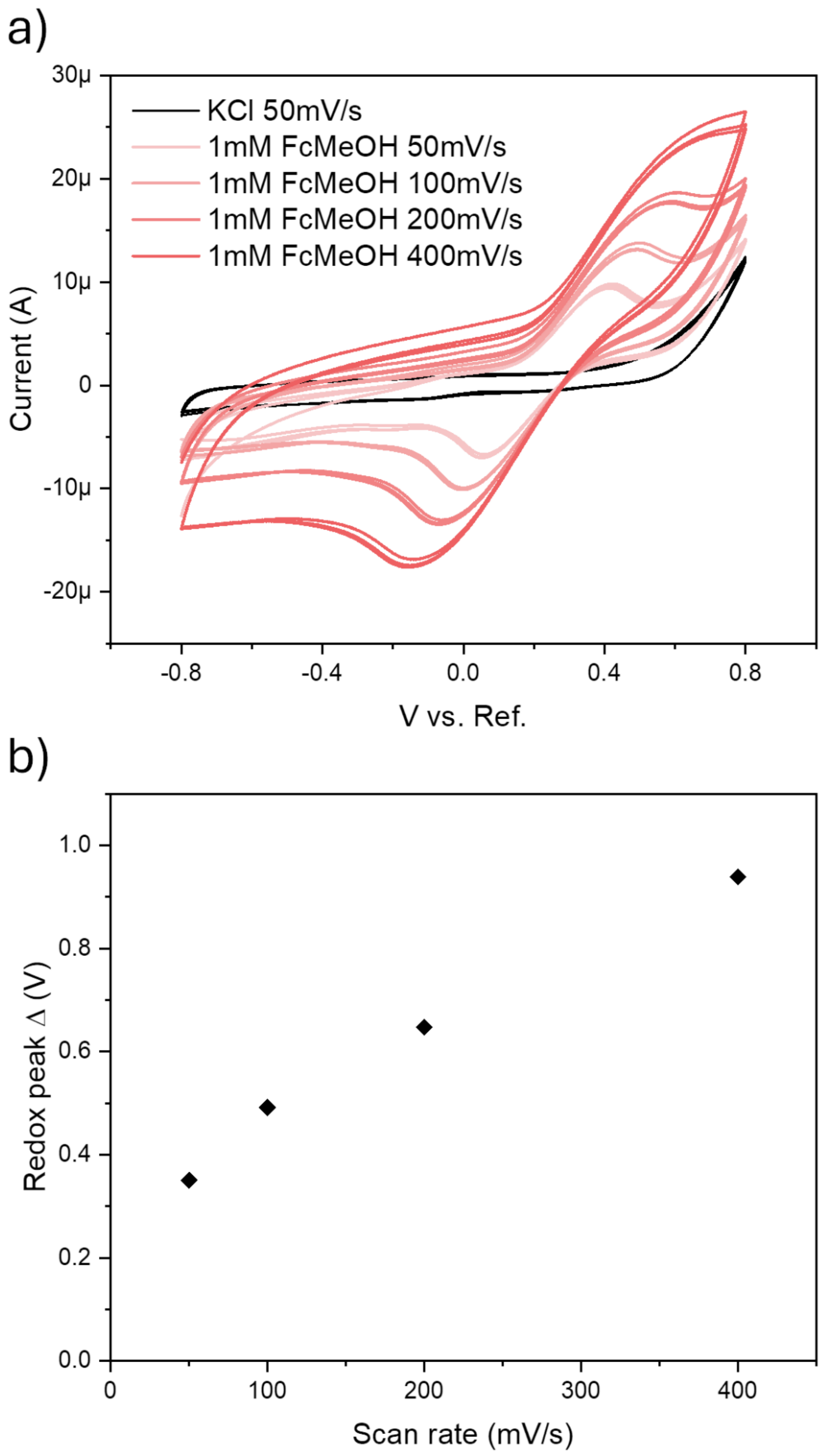

Figure S7: Outer-sphere redox probe ferrocenemethanol measurements with a) different scan rates and b) peak separation, measured with Gamry

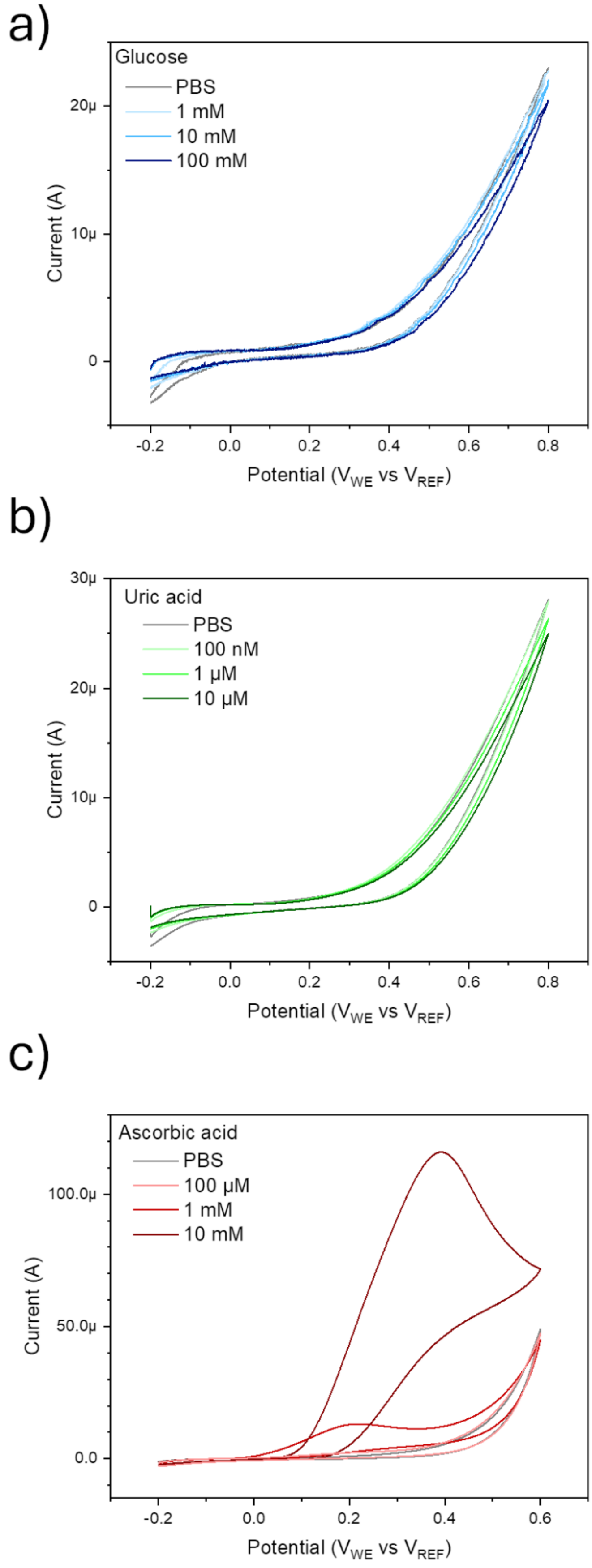

Figure S8: Selectivity measurements for a) glucose, b) uric acid and c) ascorbic acid, measured with Gamry

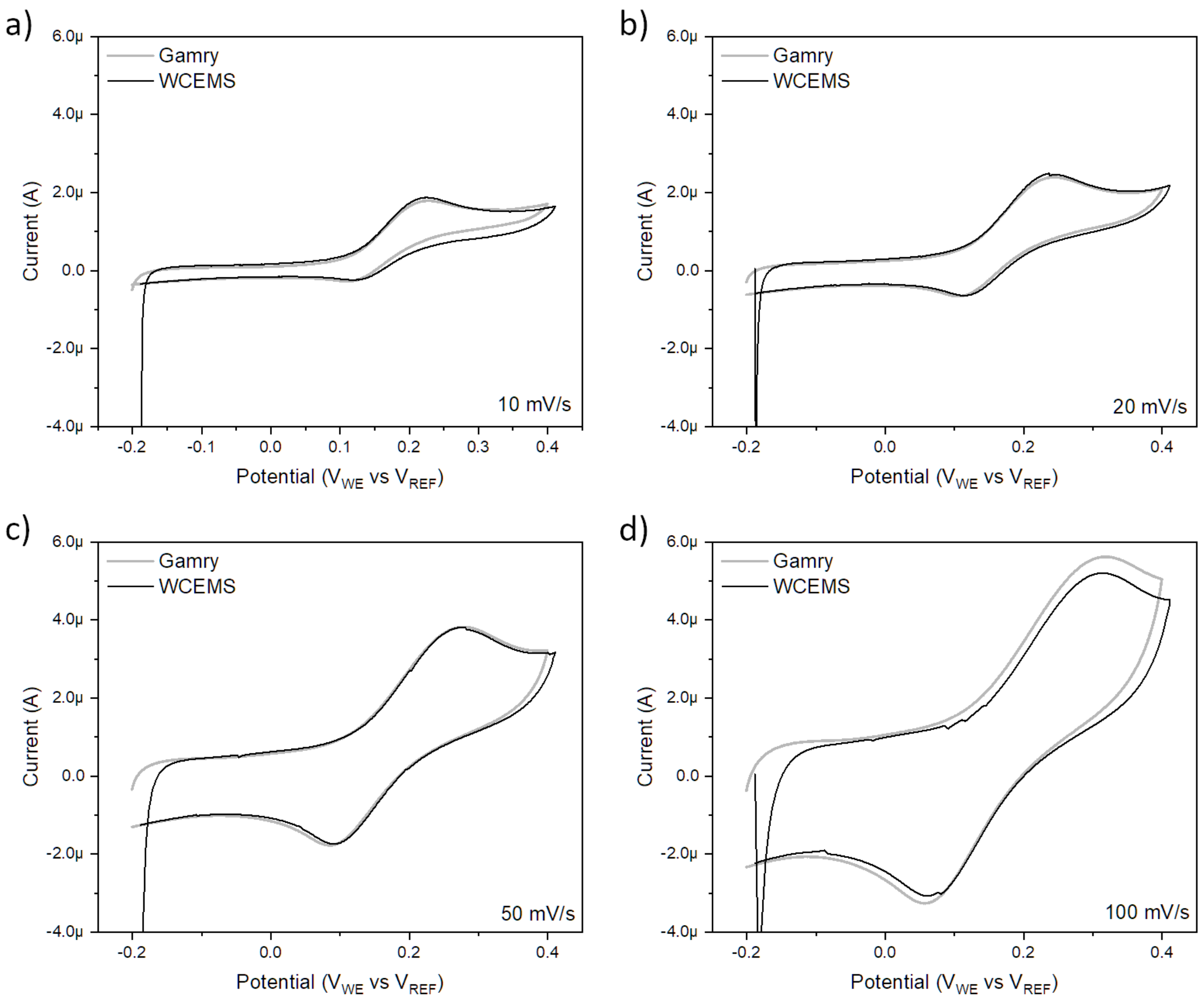

Figure S9: WCEMS and Gamry responses compared at different scan rates from 10 mV/s (a) to 100 mV/s (d)

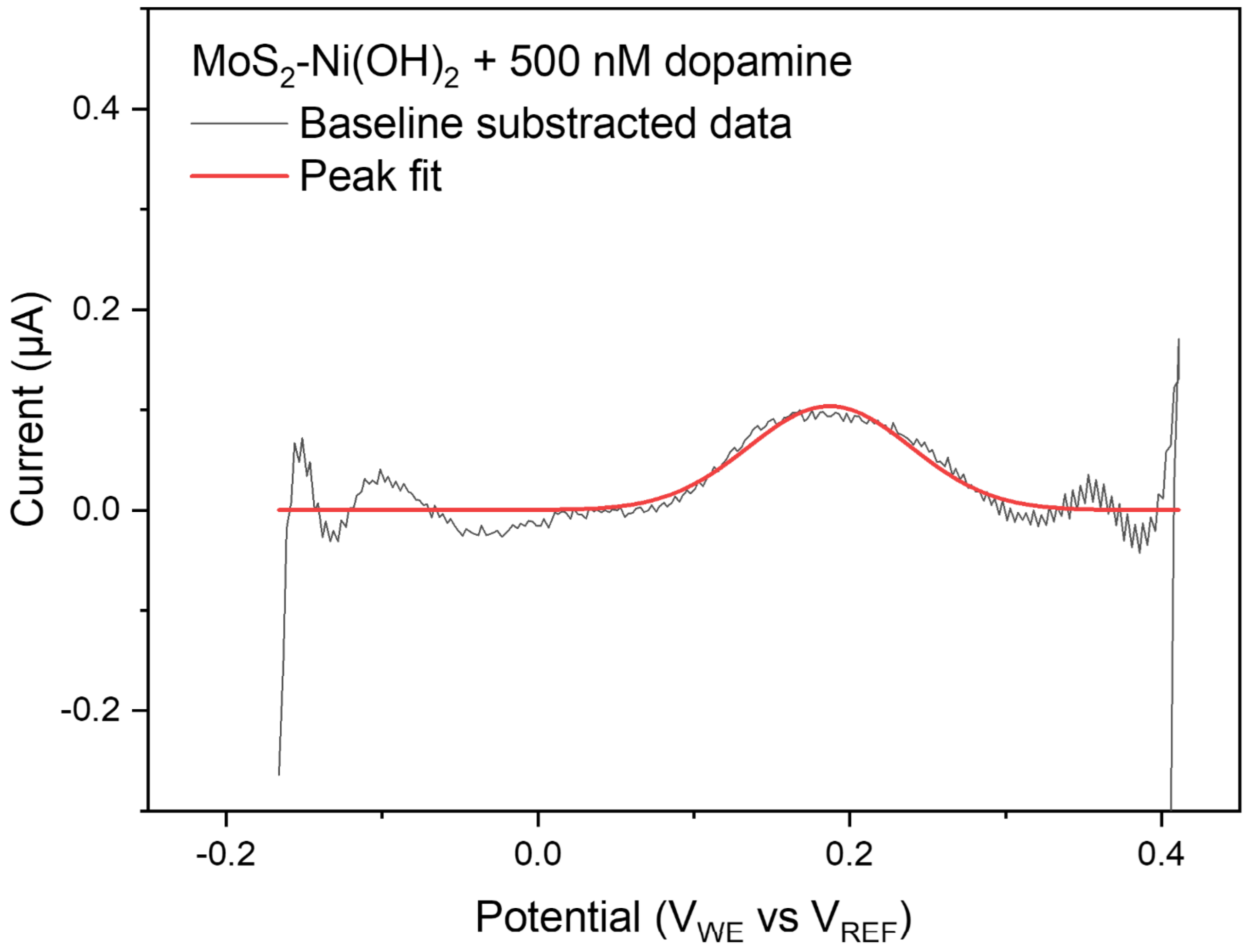

Figure S10: Peak fitting and subtracted baseline of MoS_2-$Ni(OH)_2$ sample measured by WCEMS at 500 nM concentration of dopamine

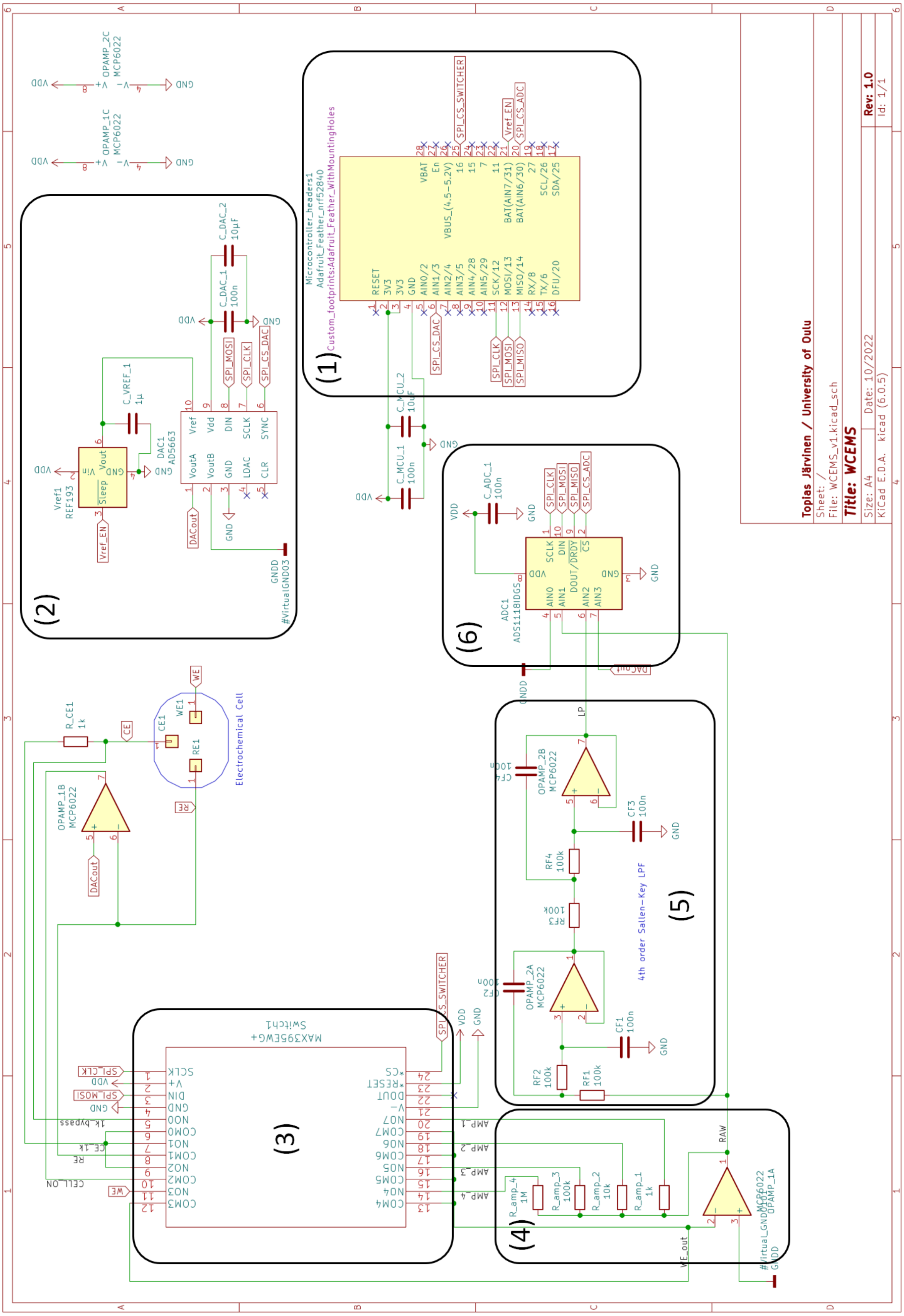

Figure S11: Schematic of the WCEMS system, consisting of (1) microcontroller, (2) DAC and voltage reference, (3) control switch, (4) transimpedance amplifier, (5) Sallen-Key low-pass filter and (6) ADC

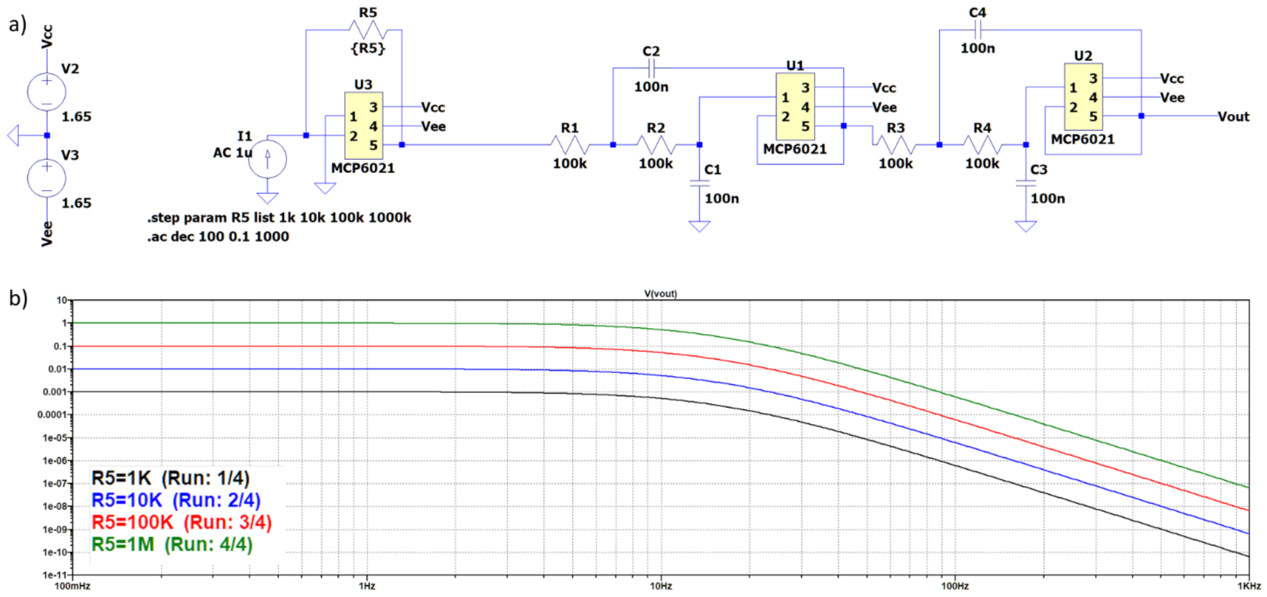

Figure S12: LTspice schematic (a) of the transimpedance amplifier stage followed by the Sallen-Key low-pass filter. Simulation for output voltage Vout (b) for an AC signal sweep between 0.1 to 1 Khz for 1 µA input current (I1) and different amplifier resistors (R5). Note: the simulation does not account for the internal resistance of switch connecting the resistors

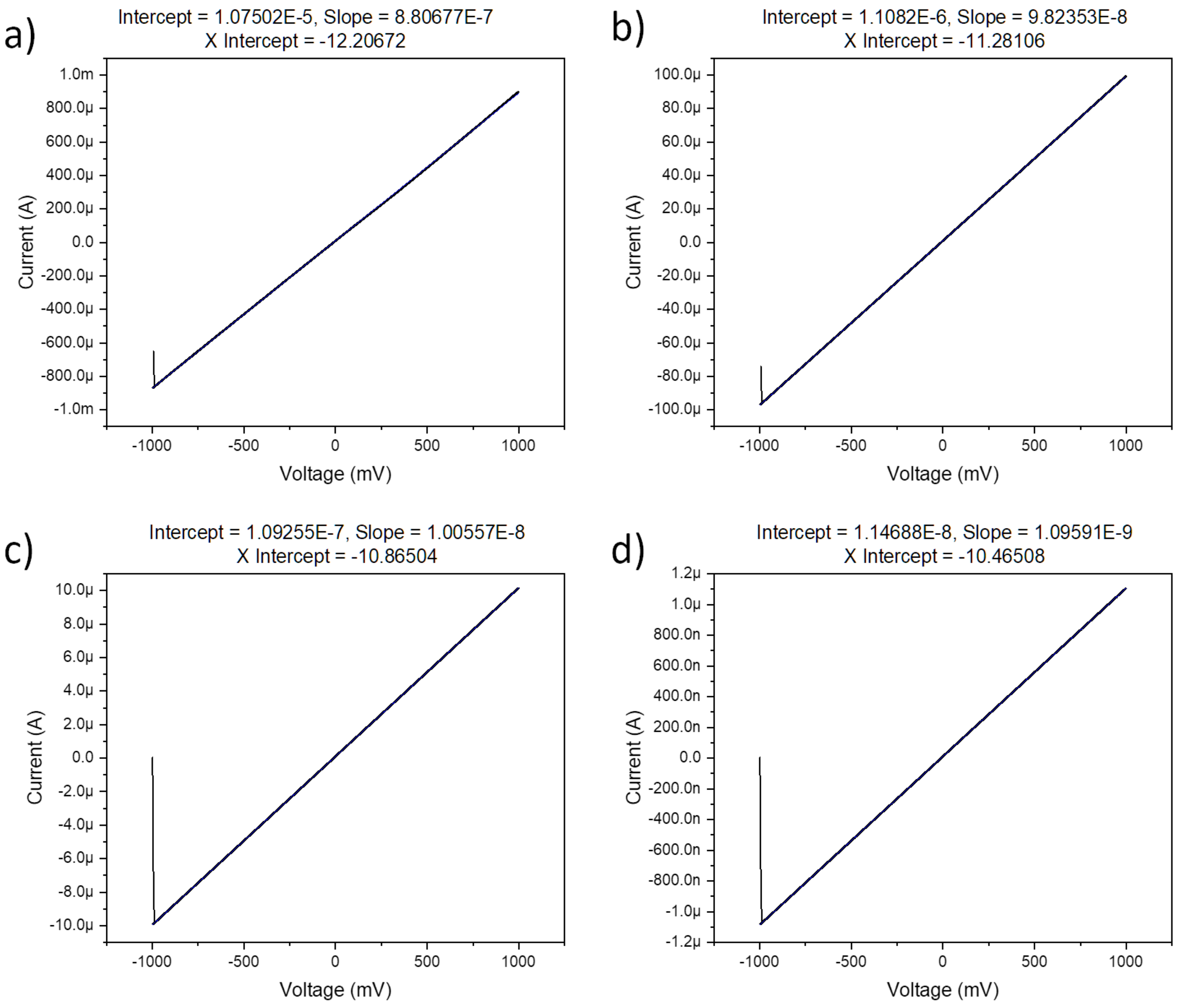

Figure S13: Calibration curves for WCEMS; a) 1 kΩ resistor with respective amplification of 10^3, b) 10 kΩ / 10^4, c) 100 kΩ / 10^5 and d) 1 MΩ / 10^6

Implementation of cyclic voltammetry functionality in the microcontroller software:

```
void CV(int startV, int stopV, int scanRate, int cycles, int amp, int electrodeConfig, int resCE){
  int vDac = 0; // raw DAC output value
  int vOut = 0; // DAC output in mV
  float vAdc = 0; // raw ADC input value
  String dataString = ""; // String for bleuart data throughput
  int mVRange = stopV-startV; // Total CV range
  int totalSteps = 200*mVRange/scanRate; // Total number of voltage steps required between voltage points, 
calculated as:
    // Refresh rate (200s^-1) * range in mV / scanRate (mV*s^-1)
  float voltageStep = (65536.0*float(mVRange))/(3000.0*float(totalSteps));
    // Single voltagestep defined by the total amount of steps and voltage range
  int initialV = midV - startV*65536/3000; // Starting point for DAC

  int j = 0; // Counter for CV loops
  int i = 0; // Counter for individual ramps
  int k = 1; // Index for datapoints

  SetSwitcher(amp,electrodeConfig,resCE); // Setting the measurement up with switcher

  for(i=1;i<=cycles;i++){
    j=0;
    vDac = initialV; // Begin loop from startV
    dac.write(CHN_1,vDac);
    while(j<=totalSteps){ // Go through voltagesteps
      currentMillis = millis();
      if(currentMillis - previousMillis >= sampleRate) { // Wait until 5 ms has occurred, then measure ADC 
and set next value
        previousMillis = currentMillis;
        if(j % 10 == 0){ // ADC samplerate 20 Hz
        vAdc = adc.getMilliVolts(); // Get ADC value
        vOut = (midV-vDac)*3000/65536; // Calculate current vOut as mV
        dataString += String(k);
        dataString += ",";
        dataString += String(vOut);
        dataString += ",";
        dataString += String(vAdc);
        bleuart.print(dataString);
        dataString = ""; // Clear datastring
        k++;
        }
        j++;
        vDac = initialV - j*voltageStep; // Set next voltagestep
        dac.write(CHN_1,vDac);
      }
    }
    // Record the peak value
    k++;
```

```
    vAdc = adc.getMilliVolts();
    dataString += String(k);
    dataString += ",";
    dataString += String(vOut);
    dataString += ",";
    dataString += String(vAdc);
    bleuart.print(dataString);
    dataString = "";

    while(j>=0){ // Return ramp
      currentMillis = millis();
      if(currentMillis - previousMillis >= sampleRate) {
        previousMillis = currentMillis;
        if(j % 10 == 0){
        vAdc = adc.getMilliVolts();
        vOut = (midV-vDac)*3000/65536;
        dataString += String(k);
        dataString += ",";
        dataString += String(vOut);
        dataString += ",";
        dataString += String(vAdc);
        bleuart.print(dataString);
        dataString = "";
        k++;
        }
        j--;
        vDac = initialV - j*voltageStep;
        dac.write(CHN_1,vDac);
      }
    }
    //Record last value
    k++;
    vAdc = adc.getMilliVolts();
    dataString += String(k);
    dataString += ",";
    dataString += String(vOut);
    dataString += ",";
    dataString += String(vAdc);
    bleuart.print(dataString);
    dataString = "";
  }

  switcherByte = 0x81; // Switch off everything, set default amplification
  switcher.write(switcherByte);

  bleuart.print("end");   // Sending end command via BLE
  Serial.println("System ready for the next measurement."); // Debug
  return;
}
```
